# How is intensive care reimbursed? A review of eight European countries

**DOI:** 10.1186/2110-5820-3-37

**Published:** 2013-11-12

**Authors:** Martin-Immanuel Bittner, Maria Donnelly, Arthur RH van Zanten, Jakob Steen Andersen, Bertrand Guidet, Jose Javier Trujillano Cabello, Shane Gardiner, Gerard Fitzpatrick, Bob Winter, Michael Joannidis, Axel Schmutz

**Affiliations:** 1 Department of Anaesthesiology and Intensive Care Medicine, University Medical Center Freiburg, Hugstetter Str. 55, Freiburg D-79106, Germany; 2 Department of Anaesthesia and Intensive Care, Tallaght Hospital, Dublin 24, Ireland; 3 Intensive & Medium Care, Gelderse Vallei Hospital, Willy Brandtlaan 10, 6716 RP Ede, Netherlands; 4 Intensive Care Unit, State University Hospital, Rigshospitalet, Blegdamsvej 9, Copenhagen, Denmark; 5 Assistance Publique, Hôpitaux de Paris, Hôpital Saint-Antoine, Service de réanimation médicale, Paris F-75012, France; 6 UPMC, Univ Paris 06, Paris, France; 7 Inserm, Unité de Recherche en Épidémiologie Systèmes d’Information et Modélisation (U707), Paris F-75012, France; 8 Medical ICU, Hospital Universitario Arnau de Vilanova de Lleida, Rovira Roure 80, Lleida 25008, Spain; 9 Adult Intensive Care Unit, Queens Medical Centre, NG11 6PE Nottingham, United Kingdom; 10 Division of Intensive Care and Emergency Medicine, Department of Internal Medicine, Medical University Innsbruck, Anichstr. 35, Innsbruck A-6020, Austria

**Keywords:** Intensive care unit, Intensive care economics, Reimbursement, DRG system

## Abstract

Reimbursement schemes in intensive care are more complex than in other areas of healthcare, due to special procedures and high care needs. Knowledge regarding the principles of functioning in other countries can lead to increased understanding and awareness of potential for improvement. This can be achieved through mutual exchange of solutions found in other countries. In this review, experts from eight European countries explain their respective intensive care unit reimbursement schemes. Important conclusions include the apparent differences in the countries’ reimbursement schemes-despite all of them originating from a DRG system-, the high degree of complexity found, and the difficulties faced in several countries when collecting the data for this collaborative work. This review has been designed to assist the intensivist clinician and researcher in understanding neighbouring countries’ approaches and in putting research into the context of a European perspective. In addition, steering committees and decision makers might find this a valuable source to compare different reimbursement schemes.

## Introduction

Over the recent years, research regarding costing and reimbursement have gained growing appreciation within the field of intensive care. One reason may be that intensive care units (ICUs) are considered to be the most expensive departmental structures in hospitals [1]. High costs of personnel, complex procedures and expensive medical devices, equipment, and infrastructure contribute to this fact [2-4]. Intensive care costs play an important role in hospital economics—both for the respective intensive care unit, the hospital it belongs to, and the healthcare system eventually reimbursing the costs.

Several studies have analysed the generation of costs in ICUs, partly also comparing procedures in different countries [5,6]. However, to date there is no widely accessible information in a scientific setting about how costs are reimbursed in the ICU setting in different countries. However, the adequate reimbursement of costs is of paramount importance for ICUs.

Therefore, national experts for ICU reimbursement and costing issues were contacted and asked for collaboration (see Table 1 for the original questionnaire). The primary goal of this collaborative effort was to give a comprehensive overview about how reimbursement works in a selection of European countries. A diversified convenience sample of eight European countries has been chosen to represent the differences in the European ICU setting. The inclusion of national experts was the key to be able to identify and explain the national systems, in many cases only rendered possible through their personal experience in the respective country’s system (thereby not even taking into account language barriers). The information generated by this amalgamation of different countries’ perspectives can be used to enhance mutual knowledge about problems faced and approaches found elsewhere (see Table 2 for a general overview on the countries’ healthcare systems).

**Table 1:** Original questionnaire used to inform all authors about uniform requirements

Health system – key facts	- Principal mode of financing (e.g., tax-based, insurance-based)
	- Number of patients admitted to hospitals per year (country-wide)
ICUs – key facts	- Number of patients admitted to ICUs per year (country-wide)
	- Number of ICUs (country-wide)
Reimbursement scheme	Please describe in detail, how ICU costs are being measured and how the reimbursement is being calculated; please refer to the clinical routine, as used in daily work:
	- Necessary documentation (is there extra documentation for budgeting purposes, or is the standard clinical documentation used?)
	- Coding (e.g., in a DRG-based system, where reimbursement is linked to diagnosis)
	- Are there differences concerning reimbursement of surgical vs. medical intensive care unit patients
	- Are there differences concerning reimbursement schemes for teaching hospitals and non-teaching hospitals (teaching refers to the education of physicians)
	- Possible modifiers (e.g., when a patient has to receive expensive medication, develops complications etc.)
	- What are, in your opinion, the most important advantages and disadvantages of your reimbursement scheme
	- Personal opinion: please explain, if you perceive a major imbalance between costs and reimbursement, i.e., if the reimbursement scheme does not adequately reflect the necessary clinical care
References	Please give references for the statements made; please feel free to include additional study results into the personal opinion part (e.g., a study conducted in your country validating your opinion or adding a crucial point)

**Table 2:** Overview: key data regarding the healthcare system and intensive care units in the countries covered in this review

**Country**	**Population**	**Healthcare system**	**Number of hospitals with ICUs**	**ICU beds per 100,000 of population**	**Number of ICU beds**	**Pts. per year (hospitals)**	**Pts. per year (ICUs)**	**Pts. hospital/Pts. ICUs**	**Average cost of ICU bed per day in Euro**	**Difficulty to find information**
Germany [7]	82 Mio	Insurance-based (statutory health insurance 90%, private medical insurance 10%)	1260	31.8	25,500	17 Mio	2 Mio.	12%	1092	Yes
Ireland [8]	4.6 Mio	Tax-based	28	5,4	250	580,000	30,000	5%	2205^b^	Yes
UK [9]	62 Mio	Tax-based	290^a^	7.5	4,700	17 Mio	200,000	1%	1500	No
Netherlands [10,11]	16.7 Mio	Insurance-based	94	9.3	1,600	1.9 Mio	70,000	4%	1290	Yes
Austria [12]	8.4 Mio	Insurance-based	132	27	2,300	2.8 Mio	-	-	2000^c^	No
Denmark [13,14]	5.4 Mio	Tax-based	49^a^	7.5	400	1.1 Mio	33,000	3%	3302^c^	No
France [15]	65 Mio	Insurance-based (statutory health insurance)	238	11.2	7,300	17 Mio	200,000	1%	-	Yes
Spain [16,17]	47 Mio	Tax-based	300^a^	7.4	3,500	5.3 Mio	240,000	5%	900 to 2500	Yes

^a^Total number of ICUs.

^b^Only known for 1 hospital, AMNCH Tallaght.

^c^for category 3 ICUs.

This also can be valuable for informing policymakers, directly influencing amendments and corrections to the systems currently used. It has to be highlighted that the differing reimbursement schemes employed in European ICUs also directly affect healthcare costs. We hope that this overview is to be seen as a valuable tool for other researchers working in the field of ICU cost-reporting and cost-generation, who might find it useful to place their findings into a European context. In the following sections, the national systems will be explained by the respective national expert.

## Review

### Germany

The German reimbursement scheme is in general based on a DRG system (diagnosis-related groups). The basic concept is the combination of a main diagnosis derived from the ICD-10 (International Statistical Classification of Diseases and Related Health Problems 10^th^ Revision) catalogue and secondary diagnoses as well as procedures listed in the OPS-301 (*Operationen- und Prozedurenschluessel* = operations and procedures classification) catalogue to form a basic DRG code. This basic DRG code can be modified according to the Patient Clinical Complexity Level, yielding the final DRG code which is reimbursed [18]. Compared with the original version, the System Version 2010 included many new features dealing with the special needs of ICUs. The 2010 system was made substantially more complex with the aim of improving the correlation between costs and reimbursements in the intensive care setting [19]. Key components of the German reimbursement scheme in the ICU setting include the possibility of varying existing diagnoses by making amendments which specify the individual patient’s health status.

The first specification is the length of mechanical ventilation. It can be coded in intervals starting with a minimum length of 96 h.

The second is the so-called intensive care complex treatment. This is an additional feature which is bound to prerequisites, such as continuous physician’s attendance and a patient’s minimum stay on the ICU of 24 h, and is formed by adding point values for special efforts in care. These point values are a combination of a daily assessment incorporating the New Simplified Acute Physiology Score (equal to SAPS II without Glasgow Coma Scale) and an assessment of ten daily activities from the TISS-28 (Therapeutic Intervention Scoring System) catalogue.

The third specification involves complicating procedures. These may include blood products, chemotherapy, central venous catheters, or pacemakers and also additional diagnoses, such as severe inflammatory response syndrome, which are combined in a multifactor-approach.

Together, these three specifications allow for a much greater variability of ICU cost reimbursement. Therefore, high standards of documentation have to be maintained. In addition to the standard documentation of clinical parameters and procedures, it also is necessary to address the features introduced above. This means, the relevant scores have to be administered and the fulfilment of other prerequisites has to be controlled. There is no difference concerning reimbursement of surgical versus medical intensive care unit patients. There also are no differences concerning reimbursement schemes for teaching hospitals versus nonteaching hospitals. However, the costs of ICUs in bigger hospitals appear to be higher than in smaller hospitals, which is probably due to a higher proportion of more cost-intensive surgical patients [20].

The increasing complexity of the German ICU reimbursement scheme requires a demanding amount of documentation and coding. This may be seen as a trade-off for a potentially higher accuracy of cost portrayal.

The system is subject to regular updates: Each year, an updated catalogue of billable DRGs is prepared, based on performance and cost data from voluntarily participating so-called "calculation hospitals".

### Ireland

Public hospitals are funded using block grant historical budgets, i.e., the previous year’s baseline allocation is generally rolled forward into the following year and then adjusted for in year national economic factors. Inherent in the funding model is the assumption that hospitals will generate a level of private health insurance income that will reduce the absolute funding amount required from the Health Service Executive to deliver services. Modifications to historical budgets generally include any nationally agreed changes to Department of Health pay scales, inflation, changes in taxes, and top slicing efficiency/"value for money" targets.

Each department in a hospital functions as a costing centre. It has fixed costs, such as amenities and staff wages, etc., and variable costs, such as medications and equipment. The fully absorbed overall costs are averaged to patient bed days, and thus a cost is assigned to a patient.

The cost-effectiveness is based on a DRG system, which derives diagnosis from the ICD-10 catalogue. All patients are coded by trained clinical coders using a chart review. Standard clinical documentation is largely used for coding purposes, although local arrangements may exist in some hospitals to enhance accuracy of coding (e.g., a special discharge summary may be completed by the ICU and or admitting teams to facilitate coding by coders). It is notable that coding, and hence costing, is currently done retrospectively, i.e., following discharge of the patient.

The coding will then generate a DRG that is assigned a relative value, which will then provide a cost, the average cost of a patient with that diagnosis. This is compared to the actual cost of a patient and a casemix adjustment is derived. Hospitals are compared to each other nationally, but they are divided into groups with large teaching hospitals in group 1, smaller hospitals in group 2, and stand-alone paediatric hospitals in group 3. Based on the casemix adjustment per hospital per group, the fixed budget can then be rewarded or penalised depending on performance. Therefore, the DRG coding is used as a performance indicator and not directly for reimbursement. There are no specific DRG codes for common ICU diagnoses, such as acute respiratory distress syndrome, multiple organ failure, or severe sepsis, which are primarily physiological diagnoses. Costly ICU treatments are primarily directed at correcting acute physiological abnormalities.

The actual reimbursement of ICU is not direct, because the ICU also functions as a costing centre. The patient bed days are divided up amongst the varying specialities with patients in the ICU. The percentage of bed days is then worked out per speciality and thus the speciality accrues that percentage of the total cost of ICU, which then comes out of the budget of that speciality.

There is no difference between medical and surgical patients as regards to reimbursement nor are there differences in reimbursement schemes for teaching hospitals versus nonteaching hospitals (except for the groups named above). Comparing ICUs from the different types of centres is difficult as more complex patients are generally cared for in the larger hospitals.

Possible modifiers for more expensive treatments (a "special costing submission") can be submitted to the Health Service Executive. This is assessed by the Health Service Executive and considered for inclusion in the following year’s budget. National specialty considerations also can be applied for if appropriate. Both the shape and structure of critical care delivery as well as the mechanism of funding of hospitals is under review with aims for profound changes (moving to a more patient-level costing system, more accurately reflecting the cost of a patient’s intensive care and hospital stay).

### United Kingdom

General funding revolves around a system of activity based funding known as Payment by Results [21]. Funding is calculated separately from the admission diagnosis funding, which is based on organ support derived healthcare resource groups (HRG). Each patient will have an admission HRG and then a separately derived critical care HRG also applies. Each critical care admission episode (spell) has a critical care minimum data set HRG calculated on the basis of the total number of organs supported during the patients stay [22]. This then provides a day rate, which is multiplied by the duration of the spell to calculate a total cost/reimbursement for the episode.

The critical care minimum data set is part of the hospital episode statistics dataset, which is held by the National Health Service information authority. This data is then returned to the hospitals via the secondary user service for sense checking. A grouper software is used to derive the HRG from the organ support data supplied.

Since 2011, the currency (i.e., the HRG) has been nationally mandated; however, the tariff (payment) has been locally set. Yet, national reference costs are generated from hospitals using annually updated guidance, thereby acting as a benchmark [23,24].

There is no difference in the reimbursement between larger and smaller units, medical versus surgical units, or teaching versus nonteaching hospitals. Some treatments, such as haematological drugs, are excluded from the payment by results system and reimbursed separately. At the moment, there is no information accessible regarding changes in the system currently in use.

### Netherlands

The reimbursement scheme is based on a DRG system, which has been introduced in 2005 and since then is being revised regularly. The number of DRGs has been reduced from 30,000 to 4,400 grouped DRGs in 2012 and the system is based on ICD-10 diagnoses [25].

Activity-based costing studies in ICUs demonstrated that time for patient care and costs were poorly associated with diagnosis but better reflected by staffing patterns, ICU levels, and a number of cost drivers, such as admission process, ICU length of stay, (non)invasive mechanical ventilation, haemodiafiltration, hospital consultation, and transportation (mobile intensive care) [26,27]. Intensive care costs add up to admission DRGs with special financial products for reimbursement (ICU add-ons). During ICU admission, all costs incurred (staffing, equipment, medications, disposables, laboratory testing, diagnostic procedures, and medical consultations) are components of the ICU budget and not part of the DRGs. The ICU incomes are based on add-on products and based on three ICU costing groups reflecting ICU complexity levels, arbitrarily divided into less than 1,000, 1,000 to 1999, and more than 2,000 days of mechanical ventilation per year.

Costs are reimbursed for fixed prices per treatment day (a), additional admission charge (b), (only first day), (non)invasive ventilation (c), and haemodiafiltration (d) surcharge fees and based on a normative ratio 20(a): 5(b): 4(c): 3(d) from the activity-based costing study [28,29]. Parallel to honorarium for intensivists the hospital costs are reimbursed using the same add-ons based on the three costing groups based on average hospital costs for ICU in 24 hospitals in 2006. Since then, prices have been indexed [30].

Due to the simplicity of the system, insurance companies can easily sample data from medical records to validate hospital claims for reimbursement. Analysis of combinations of DRGs and ICU add-ons may be of additional value. There are no differences concerning reimbursement of surgical versus medical intensive care unit patients. Furthermore, there are no differences concerning reimbursement schemes for teaching hospitals and nonteaching hospitals. The cost groups with higher volume of ventilated patients circumvent this aspect. Because all costs are in the ICU day price, normally no additional fees for procedures of medications are available. However, a few expensive medications, such echinocandins, can be additionally reimbursed [31]. In case of complications and prolonged length of stay, all ICU costs will be reimbursed.

In the future, intravenous cooling devices will be reflected by an additional fee. Due to trends of concentration in intensive care medicine, the system has to be updated to financially facilitate regional ICU systems in the interest of better regional ICU care.

### Austria

The Austrian health care plan distinguishes between intermediate or coronary care units and intensive care units. Intensive care units are separated into three categories ranging from category 1 to 3, with category 3 ICUs considered to provide care for the most severely ill patients. Classification into the three categories is based on average TISS-28 score generated over a year by each unit, with only TISS-28 scores higher than 16 being accounted (category 1: TISS-28 > 22; category 2: TISS-28 > 27; category 3: TISS-28 > 32). Every ICU category also is defined by several quality criteria that have to be fulfilled, such as minimal number of beds (i.e., six), nurse to patient ratio, level of specialisation required for physicians in charge of the unit, as well as for coverage during on-call hours.

ICU costs are measured as the sum of personnel costs and costs arising from consumables as well as from acquisition of new devices. Income results from reimbursement paid by the provincial hospital financing funds on basis of the LKF system (*Leistungsorientierte Krankenhausfinanzierung* = performance-based hospital reimbursement), which is an Austrian performance-related hospital financing system [32]. The LKF system is basically a modified DRG system, which has been introduced in 1997 and revised on an annular basis since then. The basic concept is the combination of main diagnoses derived from the ICD-10 catalogue and individual medical procedures (e.g., surgery, dialysis), which combine to an overall of 982 case groups. Each of these case groups is reimbursed by a certain number of LKF points. For ICU patients, extra reimbursement is calculated per ICU day, which is increasing from category 1 to category 3 units by a factor of roughly times 1.5. Additional reimbursement is generated for each defined medical procedure (e.g., bronchoscopy, ultrasound, specific antibody treatment) provided for the patients.

Documentation officially required by the ministry of health and the hospital administration are ICD-10 diagnoses and daily TISS-28 scoring, which determines ICU categorisation. Furthermore, SAPS II scoring on admission is compulsory. SAPS II scoring is used by the authorities for plausibility checks of the TISS-28 scores generated by each unit. Obviously, all medical procedures provided have to be documented. The TISS-28 scoring system favours surgical patients with its inherent bias on invasive procedures resulting in surgical units reaching higher categories as well as reimbursements [33].

Generally speaking, there are no differences in the reimbursement scheme between different types of hospital, i.e., teaching versus nonteaching hospitals. However, the final conversion for generated LKF points into Euros varies between the nine provinces of Austria and is mainly (politically) determined by provincial governments.

Also, the same reimbursement system applies to surgical, medical, neurological, or mixed intensive care units. However, reimbursement in surgical ICUs tends to be higher due to more frequent medical procedures provided to surgical patients.

A change in reimbursement as well as categorisation of ICUs will take place in 2014. The new system will be based on TISS-A and SAPS 3 [34] scoring. Additional compulsory documentation of these new scores has started in 2012 and their results will be used for definition of the new system (TISS-A score is a modified TISS-28 score with additional emphasis on mode of ventilation, (noninvasive) haemodynamic monitoring, agitation and delirium, assist devices (cardiac, pulmonary, liver), and therapeutic hypothermia [35]).

### Denmark

Developed and initiated by the Danish National Board of Health and the Danish Society of Anaesthesiology and Intensive Care, a new model for improvement of DRG registrations in the ICUs was implemented in 2004. The Danish ICU-DRG system consists of four groups reflecting progressive deterioration in organ failure [36]. A set amount is assigned to each of these groups. The rules for allocation to one of the four ICU-DRG groups are based on a combination of 42 intensive procedure-related codes restricted with specific demands for the time interval of ICU length of stay and length of mechanical ventilation [37].

A prerequisite for allocation to one of the four intensive groups is the provision of an intensive-related procedure code and admission to an intensive care unit for more than 72 hours. The purpose of the 72-hour limit is to weed out the less resource-demanding acute patients and the elective surgical intensive recovery process, as it was the Danish National Board of Health’s belief that the main hospitalization costs for these patient groups were outside the intensive care area.

The four ICU-DRG groups are:

ICU-DRG group I: Simple organ failure in one or two organs – Hospital length of stay 10 days (mean)

ICU-DRG group II: Progressive severe organ failure in one organ – Hospital length of stay 12 days (mean)

ICU-DRG group III: Progressive severe organ failure in two organs or more – Hospital length of stay 14 days (mean)

ICU-DRG group IV: Severe multi organ failure – Hospital length of stay 17 days (mean)

These four ICU-DRG groups are independent of the ICU level or category, ranging from one to three. Patients admitted to the ICU with an ICU length of stay less than 72 hours are reimbursed on the concept of a combination of a main diagnosis derived from the ICD-10 catalogue, secondary diagnoses, and procedure-related codes.

There is no difference concerning reimbursement of surgical versus medical ICU patients and no differences concerning reimbursement schemes for teaching versus nonteaching hospitals.

The ICU-DRG grading system does not contain reimbursement for patients receiving very expensive medication or for patients developing severe complications except for those expressed in one of the 42 intensive procedure-related codes. The ICU-DRG accounting system is evaluated by the Danish Ministry of Health with annual adaption of the DRG pricing by the use of national databases and cost registers.

### France

In 2002, a definition of ICU was issued by the French authorities with further details published in 2003. There was a strong recommendation for having an intermediate care unit along with the ICU.

The hospital funding through the DRGs was progressively introduced since 2004 and fully applied in 2008. The DRGs were refined in 2010 with four levels of severity mainly relying on comorbidities. The rules are revised every year (classification algorithm and tariffs) [38].

On top of DRG, French ICUs benefit from a complementary funding if the following three criteria are verified: patients treated in an official ICU fulfilling the nationwide criteria (board certified ICU physician, 24-h coverage by an ICU physician dedicated to the ICU, at least 1 nurse for 2.5 patients and 1 nurse’s help for 4 patients [39]), SAPS II > 15 and at least one specific ICU procedure performed during the ICU stay, such as mechanical ventilation, renal replacement therapy, or vasoactive drugs [38]. This extra funding accounts for 60% of the total payment of ICUs.

Some expensive drugs, such as modern antifungal treatment, immunoglobulin, and modern chemotherapy, are paid independently from DRG if several criteria are fulfilled: drug belonging to a restricted list (<100, updated every year), formal indication approval by health authority, individual prescription, and central preparation by pharmacist. For teaching hospitals, there is extra funding for the students, the innovation and research assessed through the number and quality of publications and number of ongoing trials. In addition, there are efforts to obtain extra funding from the ministry of health by scientific societies and hospital federations gathering all hospital across France to help meet the financial needs of ICUs.

### Spain

The system is characterized by its decentralized conception, i.e., the autonomous regions of the country (17 plus 2 autonomous cities) have the authority on medical issues, although the coordination between territories is ensured. In a universal health care system financed by taxes, the reimbursement is not the main objective in the costs allocation process. The calculation of the costs is used as a tool for comparison between centres and regions and as a quality control indicator of the medical care. However, it also is necessary for making the insurance dependent payments (traffic and labour accidents) and of services offered by private hospitals.

The decentralization of the health system has led to differences in the development and implementation of cost-accounting models developed by the different autonomous regions. In general, the basic model requires that all hospitals use the Minimum Basic Data Set which includes patient variables, episode, diagnosis (ICD-9), and codified procedures. This dataset is obtained from standard clinical documentation. The information is channelled through the autonomous regions and reaches the Ministry of Health, which calculates the reference weights of the DRGs. For a particular DRG, a comparison between the reference and calculated weights at each hospital can be established. The value of the weight integrates the information of the Minimum Basic Data Set with the hospital costs. Some regions use more advanced models, such as the International Refined DRG. Other regions also have implemented improvement and audit procedures of diagnostic coding, seeking to establish a common price by DRG, and use the model based on DRGs for the payment of the services offered by private hospitals [40].

In Spain, there are no differences concerning reimbursement schemes for teaching hospitals and nonteaching hospitals. There are no changes concerning the reimbursement of medical versus surgical intensive care unit patients or by the use of very expensive treatments or procedures. However, the system penalizes patients who undergo surgical procedures, assigning weights in a disproportionate way [41].

These problems have led to the search for improvement measures based on nationwide initiatives and opinions from scientific societies and private companies [42]. These measures should achieve the goal of a single reimbursement system that takes into account the diagnosis (using a proper ICU coding), patient severity (using severity scales and patient classification categories), specific ICU procedures (using scales of therapeutic interventions), and complications during hospital evolution [43].

## Conclusions

Our study presents how intensive care reimbursement works in eight European countries. The national experts’ contributions allow for clinicians to understand the mode of functioning in neighbouring countries. At the same time, researchers in the field of health care services can draw on this resource when putting their or others’ research into an international context. However, the most important use may be for those interacting with policymakers, thereby using this review as a source of information to present other countries’ systems. However, given the data presented in this work, we do not feel confident to make recommendations about the appropriateness and usefulness of certain systems. This would require a different methodology, ideally employing direct comparisons of standardized sample cases within certain countries, addressing both cost calculation and reimbursement. So far, these data do not exist for the ICU setting.

We present a sample of eight European countries, which was the result of a collaborative effort to include a variety of countries, thereby at no time making a claim to be exhaustive. In contrast, the sample was restricted by the identification of national experts, their willingness to contribute to this project and the timely realisation of the exchange between the experts (set to be 6-9 months). It has to be pointed out that the comparisons between countries within the setting of this collaborative project also face limitations. One important aspect is the underlying definition of intensive care units and intensive care patients, especially when differentiating high-dependency units, intermediate care units, or critical care units, as well as paediatric intensive care units or even contrasting medical or surgical ICUs. We cannot account for this variety of terms and definitions, which can be further complicated by different expressions in the various languages. Therefore, this work concentrated on intensive care units in the respective national definition, thereby excluding the other types of units, as much as possible. Still, considerable differences in the average ICU patient spectrum cannot be ruled out. This also may at least partly explain differences seen in the relative number of ICU beds and patients per country.

Finally, we would like to point out some key points regarding the data presented in this review. First, the variety in approaches used within the selection of eight European countries has to be highlighted. The ICU reimbursement schemes differ greatly, despite usually being based on DRG models. This also underlines the importance of the alterations made in the specific systems, which also may make it possible in the future to adapt the systems to lessons learnt in other countries. To make both similarities and differences more transparent, Table 3 summarizes the experts’ degree of agreement to statements regarding the inclusion of certain factors into the respective reimbursement schemes. This may be used for standardized comparisons.

**Table 3:** Basic mode of functioning of the respective national ICU reimbursement schemes, based on experts’ responses

**Basic modes of functioning ( "++" strongly agree, "+" agree, "0" indifferent or unknown, "-" do not agree, "--"do not agree at all)**								
Item	Grading							
	GER	IRL	UK	NETH	AUS	DEN	FRA	SPA
The reimbursement works per case (e.g., DRG-based).	++	-	+	++	++	++	+	+
The reimbursement works per ICU/hospital (e.g., share of reimbursement goes to all units involved).	--	+	-	+	+	-	0	0
There is separate reimbursement for hotel costs.	--	-	+	--	-	--	--	0
The following factors are taken into account for coding/reimbursement:								
1. Previous year’s ICU expenditure	--	-	-	--	-	+	--	+
2. Number of patients	--	-	+	+	+	0	+	+
3. Diagnosis (DRG)	++	+	+	+	+	+	+	+
4. Nursing workload scores (e.g., TISS-28, NEMS)	+	--	+	-	++	-	--	0
5. Severity of illness scores (e.g., SAPS, APACHE)	++	--	-	-	--	-	+	0
6. Length of stay	+	+	+	++	+	++	++	++
7. Level of organ support	+	--	++	++	+	++	++	+
Are there any plans for changes of the system in the near future?	+	+	0	+	+	--	0	0

Second, reimbursement in the intensive care setting often is highly complex. Therefore, it requires skilled personnel to work in this area. This also is an opportunity for both intensive care and public health research, especially in the educational setting, striving for greater transparency and understanding. 

Another interesting finding from our study is the great variety in access to information regarding national reimbursement schemes. Whereas some experts were able to build upon national guidelines or published reports, others reported that finding out about the systems in use and their respective mechanisms was a difficult and time-consuming process. Data collection was within the individual national expert’s competence, thereby taking advantage of experience in the matter as well as language competence. In the end, authors from five countries reported difficulties in finding the requested information, whereas in three countries information retrieval has been reported to be relatively easy. The data sources mostly used were national statistical data, original literature, and national healthcare reports. This is certainly an important finding, highlighting the importance of accessible information about reimbursement schemes as given through this review.

## Abbreviations

ICU: Intensive care unit; DRG: Diagnosis-related groups; ICD-10: International statistical classification of diseases and related health problems 10^th^ revision; OPS-301: *Operationen- undProzedurenschluessel* = operations and procedures classification; SAPS: Simplified acute physiology score; TISS-28: Therapeutic intervention scoring system; HRG: Healthcare resource groups; LKF: *Leistungsorientierte Krankenhausfinanzierung* = performance-based hospital reimbursement.

## Competing interests

The authors declare that they have no competing interests.

## Authors’ contributions

MIB and MD developed the idea for this manuscript and jointly planned it. MIB compiled all information and drafted the introduction and conclusions section. AS provided guidance, assisted in author recruitment and acted as senior author. MD, ARHZ, JSA, BG, JJTC, SG, GF, BW, MJ and AS provided the description of their respective countries’ ICU reimbursement schemes. They also actively participated in the development of the article and its refinement through discussions and suggestions for improvement. All authors read and approved the final manuscript.
